# Public Attitudes towards Medicinal Waste and Medicines Reuse in a ‘Free Prescription’ Healthcare System

**DOI:** 10.3390/pharmacy9020077

**Published:** 2021-04-08

**Authors:** David McRae, Abigail Gould, Rebecca Price-Davies, Jonathan Tagoe, Andrew Evans, Delyth H. James

**Affiliations:** 1 Pharmacy Department, Prince Charles Hospital, Medicines Management Directorate, Cwm Taf Morgannwg University Health Board, Merthyr Tydfil CF47 9DT, UK; 2 Cardiff School of Sport and Health Sciences, Cardiff Metropolitan University, Cardiff CF5 2YB, UK; A.Gould4@cardiffmet.ac.uk (A.G.); dhjames@cardiffmet.ac.uk (D.H.J.); 3 School of Pharmacy and Pharmaceutical Sciences, Cardiff University, Cardiff CF10 3NB, UK; PriceR@cardiff.ac.uk (R.P.-D.); TagoeJA@Cardiff.ac.uk (J.T.); 4 Health and Social Services Group, Welsh Government, Cardiff CF10 3NQ, UK; Andrew.Evans@gov.wales

**Keywords:** medicines reuse, medicinal waste, re-dispensing, re-issuing, redistribution, recycling, public views, public attitudes, medicines storage

## Abstract

This study investigates public attitudes towards medicinal waste and medicines reuse within a ‘free prescription’ healthcare system. A quantitative online survey was employed in a sample drawn from the population of Wales, where prescription medicines have been ‘free’ since 2007. Qualitative interviews informed the content of the attitude statements with categorical or ordinal response options assigned. The questionnaire was hosted on the HealthWise Wales platform for 1 year from October 2017. Of the 5584 respondents, 67.2% had at least one medicine on repeat prescription. Overall, 89.1% held strong concerns about medicinal waste. High acceptance for the reuse of prescription medicines which have been returned unused by patients to pharmacies was reported for tablets (78.7%) and capsules (75.1%) if the medicine is checked by a pharmacist first (92.4% rated essential). Concerns identified related to tampering of packs (69.2%) and the need for hygienic storage (65.4%). However, those working in healthcare had less concern about the safety of reusing medicines. The level of public acceptance for the reuse of medication was higher than previously reported. This is the largest survey to capture these views to date, which has implications for the future design of medicines reuse schemes.

## 1. Introduction

Medicinal waste can be produced at all points in the pharmaceutical supply chain. However, it is the waste generated when prescription medicines are returned to healthcare providers by patients, many of which remain unopened with packaging intact, which has, over the last decade, received increased interest from both researchers [1,2] and mainstream British news outlets [3,4,5,6]. These returned medicines are currently prohibited from re-entering the pharmaceutical supply chain in most healthcare systems and are, consequently, destroyed. This is considered by many stakeholders to represent an unacceptable and costly waste of limited healthcare resources [7,8].

One potential solution to reduce the amount of waste is for returned medicines to be re-dispensed to other patients. This practice, which has been referred to previously as medicines reuse [9], re-dispensing [7], re-issuing [10], redistribution [8], and recycling [2] (reuse and re-dispensing are used interchangeably in this paper), is prohibited due to concerns that returned medicines may have been stored inappropriately in patients’ homes (i.e., stored at temperatures which would cause the active pharmaceutical ingredient to degrade) or that they may have been tampered with [11].

In 2015, however, our research group identified that some pharmacists—the healthcare professionals charged with ensuring the quality of medication supplied to the public—would consider re-dispensing returned medicines if certain criteria were met [8]. These criteria included incorporating newer packaging technologies (which could alert pharmacists if medicines have been stored incorrectly or tampered with) and the need for public engagement prior to any such scheme commencing, due to concerns about how the public would accept medicines reuse [8].

At the time this study was conceptualised, little research had been conducted into how the public perceive the issue of medicines reuse. In 2011, the National Health Service (NHS) Sustainability Development Unit in the United Kingdom (UK) found that 52% of a sample of 1101 people living in England would accept medicines returned by other patients if they have been checked for safety [10]. Unfortunately, no further questions were asked to elicit the details of what would be expected to be included in a safety check or whether there were any other criteria participants would require before they would accept returned medicines. Following this, Hendrick and colleagues conducted a small survey of hospital in-patients and out-patients, which found that 66% (of the 59 respondents) would accept reused medicines, but that few (specific figures not provided) would do so unconditionally [9].

Medicines reuse schemes exist in some private healthcare systems, such as Greece and the United States of America (USA), where reuse allows members of the public to access medicines which they would not otherwise be able to afford [12,13]. To date, no research had been conducted with the aim of sampling public attitudes towards medicines reuse and medicinal waste drawn solely from a healthcare system where prescription medicines are ‘free’. Some policy makers may share the belief expressed by primary care professionals, in interviews conducted by Truman and colleagues, that people receiving free medicines do not value them [14]. Anchored by that belief, policy makers may also take the view that there would be little incentive for the public to accept medicines reuse (in a free medicines healthcare system) and that concerns about medicinal waste would be low.

The aim of this study was, therefore, to determine public attitudes towards medicinal waste and medicines reuse in a large sample of the general population, within a ‘free prescription medicine’ healthcare system, through the use of a web-based platform where members of the public register to receive health related questionnaires. This study sought to identify whether the public has concerns about the reuse of medicines and what criteria, if any, would need to be met for medicines reuse to be accepted. In addition, the suitability of returned medicines for reuse may depend on how they have been stored and, therefore, this study also aimed to capture information on domiciliary medicines storage practices. Furthermore, it has been suggested that to sustain a reuse scheme the public would need to be encouraged to return their medicines to healthcare facilities [15,16]. Previous research, conducted in 2006, found that only one in three respondents disposed of their medicines appropriately (by returning to healthcare facilities) [17] and as such, this study also seeks to establish current medicines disposal practices of the public and whether the public would be more likely (or not) to return medicines to pharmacies if medicines started to be reused. This study was predominantly exploratory in nature. However, one hypothesis was tested, which arose from previous research relating to pharmacists’ positive views about a re-dispensing scheme [8]. Our hypothesis was:

Healthcare professionals hold stronger concerns about medicinal waste and less perceived concerns about the safety of medicines reuse than non-healthcare workers.

## 2. Materials and Methods

### 2.1. Overview of Study

A predominately quantitative approach was adopted to meet the aims of this study. A questionnaire was developed and hosted on a web-based platform (HealthWise Wales, Cardiff, UK). The questionnaire was designed to capture participants’ attitudes towards medicinal waste, medicines reuse and to identify contemporary domiciliary medicines storage and disposal practices.

### 2.2. Study Setting

Wales is a country in the UK with a population of 3.1 million people [18]. Prescription charges were abolished by the Welsh Assembly Government in April 2007 [19]. Prior to this, prescriptions were only free for people under 25 and over 60 years of age or for those who had certain medical conditions [19].

The questionnaire was hosted on the HealthWise Wales’ platform which is the survey’s virtual online interface comprising the HealthWise Wales Website and Web Application (data collection tool) [20]. People over the age of sixteen years of age living in Wales or using health services in Wales have been eligible to register with the HealthWise Wales platform since it was launched in 2016. Registered platform users are contacted every six months and asked to complete a suite of questions of which the present questionnaire was one.

### 2.3. Data Collection

HealthWise Wales participants provide demographic data (see Table 1) as part of a core module when registering with the platform. Educational attainment is not collected. Employment status is measured using a 4-category classification of employment (higher occupations; intermediate occupations; lower occupations; students or long-term unemployment).

The Welsh Index of Multiple Deprivation (WIMD), the Welsh Government’s official measure of relative deprivation, is determined for each HealthWise Wales participant from the address entered when registering. Deprivation, within the WIMD, refers to the degree to which the needs associated with each indicator (income, employment, health, education, access to services, housing, community safety and physical environment) are met [24]. All areas in Wales (*n* = 1909, average population of 1600) are ranked from 1 (most deprived) to 1909 (least deprived) [20]. Areas are then divided into five relative deprivation categories (or quintiles): 10% most deprived (areas 1–191); 10–20% most deprived (area 192–382); 20–30% (areas 383–573); 30–50% (areas 574–955) and 50% least deprived (areas 956–1909) [24].

### 2.4. Questionnaire Design

Qualitative interview data from an MPharm undergraduate student project [JT] conducted in 2014 informed the content of the questionnaire. The questionnaire items were developed following analysis of the transcripts of eleven interviews conducted to gather views on medicinal waste and medicines reuse with members of the public between 30 and 70 years of age recruited via GP practices in one healthcare authority in Wales. All interview participants were in receipt of repeat medications from the GP surgery. Content analysis of 32 comments made by the general public under a web article about the potential for reuse of medicines [25] was also utilised. The questionnaire was subsequently piloted on a convenience sample of ten members of the public from South East Wales and community pharmacy users from South West Wales. Following feedback, several questions were removed in an attempt to improve face and content validity (these questions focused on the role of medicines cost to the NHS) and a definition of regular medicines use added (those medicines which are on ‘repeat prescription’). The resultant questionnaire (Supplementary Materials Data S1) comprised twelve questions (question 11 having two parts).

Question 1 asked participants whether they considered themselves to work in healthcare, with question 2 providing a list of healthcare roles to choose from for those answering in the affirmative to question 1.

Question 3 sought to determine whether participants were prescribed medication regularly (on repeat prescription).

Question 4 aimed to determine whether respondents were concerned about ‘the amount of prescription medicines’ wasted in the healthcare system. Respondents were asked to rate their agreement on a 5-point Likert scale (Strongly Agree, Agree, Neither Agree or Disagree, Disagree, Strongly Disagree) plus a response option for Don’t Know.

Question 5 asked respondents where they stored medicines within their home. Respondents were able to select one or more location from a pre-generated list (Living room, Kitchen, Bathroom, Entrance hall, Other, I don’t have medicines). If ‘Other’ was selected, participants were provided with space to enter the location.

Question 6 asked participants what they did with medicines they no longer needed. Participants were provided with a list of possible ways of dealing with medicines which they no longer needed (throw out with household waste, keep just in case I need in future, return to pharmacy, return to GP, I don’t use medicines or other) from which they were able to select more than one option. If ‘Other’ was selected, participants were provided with space to enter how they dealt with these medicines.

Question 7 asked participants what they believed currently happened with medicines that were returned to pharmacies. Participants were provided with a list of options from which they could choose one (re-dispensed to other people, sent to developing countries (or ‘third world’), destroyed, not sure, other). If ‘Other’ was selected, participants were provided with space to enter what they believed happened to these medicines.

Question 8 was presented as a table that included a list of pharmaceutical forms. Participants were asked which of the types of medicine they would accept if they were re-dispensed. Participants were able to select “yes”, “no” or “unsure” for each form.

Question 9 was presented as a table that included statements that sought to determine factors (or conditions) that participants would need to be in place for them to accept a medicine that had been returned to a pharmacy by someone else. Participants were able to select a response of: ‘Essential’, ‘Desirable’, ‘Unsure’ or ‘Not Needed’, for each statement.

Question 10 was presented as a table that included statements which sought to determine participant’s beliefs about the safety of reused medicines and concerns about reuse schemes. Participants were asked to indicate their agreement (or disagreement) with each statement on a 5-point Likert scale (Strongly Agree, Agree, Neither Agree or Disagree, Disagree, Strongly Disagree). Participants were also provided with a ‘Don’t Know’ option.

Question 11 had two parts. Part A asked participants whether they would be more or less likely to return medicines that they no longer needed to a pharmacy if medicines started to be reused. Participants could choose one of the following statements “More likely to return to a pharmacy”, “Less likely to return to a pharmacy” or “Would not change how I get rid of medicines”. Part B asked how participants return their unused medicines to a pharmacy. Participants were provided with the following options to select from: ‘Always’, ‘Often’, ‘Sometimes’, ’Rarely’ or ‘Never’.

Question 12 asked participants whether they thought all medicines should be considered for re-dispensing or only those which were expensive. Participants were able to select from one of the following options: ‘Only expensive medicines (perhaps costing the NHS greater than £20) should be considered for re-dispensing’, ‘all medicines should be considered for re-dispensing’, or ‘not sure’.

### 2.5. Sampling and Recruitment

The questionnaire was made available on the HealthWise Wales platform between October 2017 and October 2018. The number of registered users in October 2017 was 12,818, and 26,198 in October 2018. Due to the dynamic nature of the number of platform users, it was not possible to calculate a response rate for this study.

### 2.6. Analysis

The data were accessed and analysed via the HealthWise Wales Information Repository (SAPPHIRe), which is implemented on the UK Secure eResearch Platform (UKSeRP) [26] using IBM Statistical Package for Social Sciences (SPSS) version 26 ©.

Basic descriptive statistical analyses were undertaken for the demographic characteristics, i.e., gender, age, ethnicity, health board, level of employment, level of deprivation, and whether they worked in healthcare. Categorical data such as participant storage practices, disposal of medicines, current fate of medicines returned to the pharmacy and level of acceptance for twelve formulations of medicines considered for re-dispensing were summed and percentages calculated. Frequency distributions were calculated for Likert scale responses for the ‘concerns about medicinal waste’ item, nine ‘factors affecting acceptance for reuse’ items (Question 9) and nine items to measure ‘concerns about the safety of re-dispensing prescribed medicines’ (Question 10).

#### 2.6.1. Factor Analysis, Cronbach’s Alpha and Scale Score Analysis

All items relating to ‘beliefs about the safety of medicines reuse’ were negatively worded apart from one—‘It is safe for other people to use medicines that I have returned’ (Q10b). This item was reverse scored prior to analysis to ensure that 5-point Likert scale response were in the same direction as all other items. ‘Don’t know’ responses were treated as missing data and removed from the analysis. Principal Component Factor Analysis was conducted for all nine items using Varimax Rotation with Kaiser Normalisation and the Eigen value was set to 1 [27]. This yielded a two-component matrix where 59.8% of the variance was explained by two factors (see Supplementary Materials Data S2). For the purpose of this analysis one 5-item scale (Q10a, b, d, e, f) was used to represent concerns about the safety of medicines reuse and this was labelled ‘perceived safety of medicines reuse’. Cronbach’s alpha analysis was undertaken to check for the internal consistency of the scale. A Cronbach’s alpha value above 0.70 is acceptable [28] and in this case was calculated as 0.817 indicating excellent internal reliability. Individual item scores were therefore summed to produce a total scale score for ‘perceived safety of medicines reuse’ with a minimum possible score of 5 and maximum possible score of 25 and mid-point of the scale of 15. Higher scores indicate stronger beliefs that the concept of medicines reuse is unsafe.

#### 2.6.2. Relationships with Beliefs about Safety of Medicines Reuse Scale Scores

Parametric tests such as independent sample *t*-tests and one-way Analysis of Variance (ANOVA) were used to test for statistically significant differences between scale scores which were normally distributed (e.g., perceived safety of medicines reuse scale) and dichotomous variables (e.g., healthcare vs. non-healthcare worker). Non-parametric tests (i.e., Mann–Whitney) were used to test for statistically significant differences in scores where data were not normally distributed (e.g., concerns for medicinal waste) and dichotomous variables (i.e., healthcare vs. non-healthcare workers). A probability level of *p* < 0.05 was set as a benchmark for reaching statistical significance.

## 3. Results

### 3.1. Participants

Table 2 summarises the participant demographic characteristics. Of the 5584 HealthWise Wales members who completed the questionnaire, over two-thirds were female. Participants’ age ranged from 16 to 96 years (mean age = 53.1 years; SD = 16.059).

One-fifth of participants (19.9%, *n* = 1109/5584) indicated that they worked in a healthcare setting, with nearly half (47.9%) working in non-clinical roles and 52.1% in patient-facing roles.

### 3.2. Concerns about Medicinal Waste (Question 4)

Responders reported strong concerns about medicinal waste (mean score = 4.46; SD = 0.719), where 89.1% strongly agreed or agreed with the statement shown in Table 3 and therefore these scores were not normally distributed.

### 3.3. Medicines Storage and Disposal (Questions 5–7)

Table 4 details participants’ storage and disposal practices. The majority of participants reported keeping their prescribed medicines in either the kitchen, bedroom or bathroom. Over half said they returned unwanted medicines to the pharmacy and over one-quarter reported keeping unused medicines for the future. Over three-quarters of participants were aware that returned, unused medicines are currently destroyed.

### 3.4. Views about Medicines Reuse (Question 8)

Table 5 reports the formulation of medicines which the participants would be prepared to accept if they were re-dispensed, indicating that tablets and capsules have the highest acceptance for reuse.

### 3.5. Attitudes towards the Possible Reuse of Medicines (Questions 9)

Table 6 summarises factors affecting the acceptance of a medicines reuse scheme. The most important factor was the checking of the medicine by a pharmacist, with this being reported as the most important criterion. The next most essential factors were that the medicine is still in date, that the medicine has been returned unopened and with an intact tamper proof seal to confirm that they were unopened.

With regards to whether the cost of the medicine should dictate which medicines are considered for re-dispensing, the majority said that all medicines should be considered (79.5% agreement), not only the expensive ones (7.6% disagreement, 12.8% not sure). Respondents indicated that they would be more likely to return unused medicines to the pharmacy if a re-dispensing scheme were initiated (Table 7).

A total of 2531 respondents reported that they do not return their unused prescriptions medicines to the pharmacy. Of these, 84.5% (*n* = 2128/2519 who answered the question) indicated that they were more likely to return their medicines to a pharmacy in the future if a medicine reuse scheme was in place. Only 1.9% said they would be less likely to return medicines to the pharmacy and 13.6% would not change their current practice of disposing medicines.

Table 8 presents participants’ concerns about the safety of introducing a re-dispensing scheme. The strongest concern related to the possibility that returned medicine packs could have been tampered with or that they may not have been stored hygienically. In contrast, most agreed that medicines they had returned themselves would be safe for others to use.

Scale scores for the 5-item scale ‘perceived concerns about the safety of medicines reuse’ ranged from 5 to 25, utilising the full range of possible scale scores and were normally distributed (median = 17.0; mean = 16.2, SD = 4.359, *n* = 5383) with 36.7 scoring up to and including the mid-point of the scale (MP = 15). A higher scale score indicated less concern about the safety of medicine reuse suggesting that the majority of respondents considered the reuse of medicines to be safe.

Differences in Healthcare Professionals’ Concerns about Medicinal Waste and Perceived Safety of Reuse.

Hypothesis: Healthcare professionals hold stronger concerns about medicinal waste and less perceived concerns about the safety of medicines reuse than non-healthcare workers.

Healthcare workers reported a significantly more concern for medicinal waste than non-healthcare participants (U = −6.937, *n* = 5455, *p* < 0.001). There was a significant difference in ‘perceived safety concerns for reusing medicines’ scale scores for those who worked in healthcare (mean = 16.57, SD = 4.315, *n* = 1085) and those who did not (mean = 16.11, SD = 4.365, *n* = 4257) (mean difference = −0.462, df = 5340, *p* < 0.01), with those working in healthcare being less concerned about the safety of a medicines reuse scheme.

## 4. Discussion

This study has found a large proportion of respondents (78.7% for tablets and 75.1% for capsules), in a sample drawn entirely from a ‘free prescription’ medicines healthcare system, indicating that they would accept the reuse of oral solid pharmaceutical dosage forms. However, this acceptance is caveated by strong concerns, held by the same respondents, about the quality and safety of these medicines. The results of this study also provide support for our hypotheses that healthcare workers would have stronger concerns about medicinal waste and less concern about the safety of a medicines reuse scheme compared to individuals not working in healthcare.

This questionnaire was designed and piloted in 2016. At that point in time, little research had been undertaken into how the public viewed medicines reuse, and, of the research which had been conducted, none had sought the views of individuals residing in a ‘free prescription’ medicines healthcare system. Since 2016, the number of researchers working in the area of medicines reuse has increased and significant gains have been made in understanding the public perspective of this issue [7,29,30,31,32]. The results of this study support the findings of other researchers [7,29,30,31,32]. Additionally, when considering public attitudes towards medicines reuse in the UK, this study has provided insight into the views of the Welsh public and provides an indication of how medicines reuse may be perceived within the other ‘free prescription’ medicines healthcare systems of the Union, namely Scotland and Northern Ireland. The recruitment strategy employed in the present study was effective in that it was able to harness the views of the general public, rather than more conventional approaches to recruitment in pharmacy settings using customer surveys [33].

The hypothesis that healthcare professionals would have stronger concerns about medicinal waste and fewer concerns about medicines reuse was derived from our previous study with healthcare professionals [8]. In the qualitative interviews which informed our Delphi study, we noted strong concerns at the amount of medicinal waste amongst the healthcare professionals (doctors, nurses and pharmacists) interviewed. We also identified, through the Delphi study, that pharmacists could support re-dispensing returned medicines if certain criteria in place, which led us to believe that healthcare professionals may have fewer concerns than the general public. We speculate that the support for medicines reuse amongst healthcare professionals may be influenced, at least in part, by a desire to ease the tight budgetary conditions in which they operate (in the UK)—through saving money which they see as currently being wasted—to allow for increased spending on direct patient care. Additionally, while the proportion of respondents identifying as working in healthcare in the present study should be viewed as a limitation to our findings (discussed below), we also believe that capturing the views of so many healthcare professionals is an important finding in the field of medicines reuse research. Alhamad and colleagues, who have evaluated a Theory of Planned Behaviour-based questionnaire (their Medicines Reuse Questionnaire (MRQ)), found that the views of doctors and pharmacists would play an important role in norm-based intentions to accept reused medicines [32]. A conclusion from Alhamad’s study based on this finding being that interventions which encourage doctors and pharmacists to endorse medicines reuse being needed to help the public embrace reuse schemes. Our findings suggest such interventions would be welcomed by these professional groups.

It is of interest that the present study has found higher levels of acceptance towards medicines reuse than two other large quantitative surveys undertaken with a similar aim [29,30,32]. Alhamad and colleagues developed and validated the MRQ which was distributed to a representative sample of the UK public, drawn from its different regions [29,32]. Participants were presented with a precise definition of reuse behaviour: “*...would [you] personally consider reusing medication in the future. We define reusing medication as the idea that you would accept for your own personal use a prescription medication that has been previously given out to another patient but then returned to a pharmacy, where the pharmacist has verified that the medication: has been kept by the other patient for less than three months, has more than six months of shelf-life remaining, has not been tampered with, has been kept under normal storage conditions, and has been kept in an original sealed blister pack* (*i.e., medication strip*)*. When we refer to reusing medication, we are interested in prescribed medication that an individual/patient may use for a long-term illness. The individual/patient would be well enough to make their own healthcare decisions.*” Of the 1003 valid responses received, 54.5% ‘intended to’, 56.5% ‘wanted to’ and 56.5% ‘expected to’ reuse medicines in the future [32]. Bekker and colleagues administered a medicines reuse questionnaire to community pharmacy users in a region of the Netherlands [30]. Of the 2215 participants, 61.2% indicated that they would personally be willing to “*reuse medication returned unused to the pharmacy by another patient if the quality was guaranteed*” [30]. There are a number of possible reasons for the difference in the rates of willingness, acceptance or intention to reuse observed in this study. One reason for the difference may be the relatively high proportion of healthcare workers participating in our survey, who, we have identified, have less concern about medicines reuse than non-healthcare workers. Another potential reason could be due to Bekker and colleagues asking participants about reusing medication in general as opposed to specific pharmaceutical dosage forms [30]. As our current study and a qualitative interview study by Alhamad previously identified, the public exhibit different levels of acceptance towards medicines reuse dependent on pharmaceutical forms [31]. In their study, Alhamad and colleagues found that this preference was influenced by beliefs about the protection against tampering afforded by the more traditional packaging associated with dosage forms (‘creams come in a tube’) and beliefs about the ease with which such tampering could be to detected.

Respondents in the present study reported concerns about the quality and safety of reused medicines which have been found by other researchers working in the area [7,30,31,32]. While respondents were concerned about how hygienically medicines had been stored by others, few respondents were concerned that the reuse of medicines could spread disease or believed that medicines returned by others would be ineffective. It is of note that Alhamad and colleagues found, in their qualitative interview study, that participants had concerns about the logistics of a potential medicines reuse scheme [31]. Our focus, when designing the questionnaire for the present study, was on the quality and safety of reused medicines, but, it appears that, based on Alhamad and colleagues’ findings, that the public have concerns which extend beyond what is dispensed to them [31]. Indeed, we were surprised that over three-quarters of respondents’ number of respondents in the present study who knew that medicines returned to pharmacies were destroyed. Taken together, concerns about the logistics of a potential reuse system and awareness of the current fate of medicines returned to pharmacies, it is apparent that a proportion of the public are well informed about the issues which surround medicinal waste and medicines reuse and this should be considered when medicines reuse schemes are designed.

We have also found similar requirements of medicines reuse to other questionnaires that have been conducted in this area [30,31,32]. One exception to this was the finding that over 90% of respondents in the current study considered that a pharmacist check of the returned medicine was essential. This finding may indicate high levels of public trust in pharmacists in the healthcare system sampled.

Most respondents reported storing medicines in kitchens and bathrooms, which is contrary to guidance on the correct storage of medicines in the home [34]. It is believed that storage in kitchens and bathrooms may expose medicines to temperatures above those which manufacturers recommend and that this could lead to the medicine having reduced efficacy and an increased potential to cause side effects [35]. Providing support for this concern, Hewson found maximum temperatures in bathrooms and kitchens of 31.5 and 32.8 °C, respectively [36]. However, mean temperatures for the areas were much lower (18.4–23.6 °C) [36] and the potential for isolated or regular but transient high temperatures to negatively affect medicines, particularly when considering storage periods of 3–6 months, is disputed [15]. Indeed, when researchers have assessed all temperatures that medicines have been exposed to over a pre-determined period in the home environment, they have found that the majority of patients store their medicines in acceptable temperature conditions (both studies looking at medicines which needed to be stored either below 25 or 30 °C) [37,38]. As medicines which are sensitive to humidity are stored in protective packaging, it is temperature that remains the primary storage concern when considering medicines reuse. Researchers (including ourselves) interested in medicines reuse have proposed digital solutions [39], pointed to the existence of smart temperature labels [15] and envisaged the use of temperature monitors as part of reuse schemes [8,40] as a solution to identifying medicines which have been stored at inappropriate temperatures within homes. However, we find ourselves persuaded, in agreement with the conclusions of Mackridge and Marriot and Donyai and colleagues [41], that future work aiming to overcome the barrier which storage temperature in the home has posed towards medicines reuse should focus on improving the packaging in which medicines are supplied (by the manufacturer). It is essential, not just for medicines reuse, but for the primary recipient (from the first dispensing) that medicines are supplied in packaging that are able to tolerate reasonable domiciliary temperature conditions (including those encountered in bathrooms and kitchens) in the home.

Over half of respondents in the current study reported returning medicines to pharmacies for disposal. This contrasts with other studies which have found that only small proportions of respondents disposed of medicines in this way, with the majority disposing of medicines in household waste or via wastewater systems [17,42]. This finding may be due to greater public awareness of the harm that inappropriate medicines disposal can have on the environment or because of campaigns to increase awareness of the risk that unused medicines in the home create for accidental ingestion by children or deliberate ingestion as part of suicide attempts in the sample population. 

The present study has several limitations which must be considered when interpreting the results. Only one question about concerns about medicinal waste concern was included in the questionnaire. As with any single item measure, this approach is not robust in terms of psychometric properties and we advise caution in the interpretation of this finding.

While this study captures a large sample from the population of Wales, the sample is self-selecting in nature which has introduced sampling bias into this study. We are unable to provide assurance that non-responders (or non-registrants with the platform) would share the views of those we have captured. Females, aged between 45 and 64 years old and professional occupational class are over-represented in the Healthwise Wales population compared to the population of Wales and this was also the case in our study sample. Similarly, those over 65 years of age and in routine and manual occupational classes are under-represented in both the HealthWise Wale population and our study sample. Nevertheless, a wide range of demographic characteristics and geographical locations of Wales are represented in the findings, but the findings should not be taken as being generalisable to the population of Wales as a whole.

Proportionally, ethnic minorities are poorly represented in the sample and further work must attempt to increase participation of these groups in further research on the subject so that views form these groups can be captured. While the HealthWise Wales platform has provided us with access to a large, geographically dispersed sample with a mix of demographics (apart from minority groups) it is also important to acknowledge that a proportion of the public, 13% in Wales at the time this study was conducted, did not have home internet access and that the views of this group have also not have been captured [43].

We have also noted that the proportion of respondents identifying as working in healthcare (approximately one-fifth) is greater than the proportion of the population that work in healthcare (approximately 2%) and, as such, that the views of healthcare workers are over-represented in the findings [44]. Moreover, over two-thirds of respondents had at least one medicine on repeat prescription and therefore had some level of familiarity with the healthcare system in Wales. Proportionally, it is likely that this constitutes an over representation of repeat medicines users when compared to the population of Wales (previous estimates of the proportion of population in receipt of at least one repeat item from the UK being 43–48% [45,46]). These limitations should be considered when interpreting these findings and when applying the results to other settings.

Further research is needed to establish whether medicines that are returned to pharmacies are suitable for reuse. We see this as a sequential piece of research which would commence with a multidisciplinary panel (including pharmaceutical scientists) identifying commonly dispensed medicines which are likely to remain stable in the presence of temperature fluctuations likely to be experienced in the home. The next stage would seek to identify whether a questionnaire, designed to establish the storage conditions a returned medicine has been exposed to, administered at the point of a medicine being returned to pharmacy, could be validated to identify medicines which are suitable for reuse (through the pharmaceutical analysis of returned medicines).

## 5. Conclusions

A growing body of research is highlighting that the majority of the general public would favourably receive reused medicines for their personal use. This study contributes to how medicines reuse is viewed amongst a large sample of the public from a ‘free prescription’ healthcare system. Importantly, it also contributes that medicines reuse appears to be supported by healthcare professionals, whose views on the matter would play a significant role in influencing the general public’s attitudes towards reuse when it becomes a reality.

## Figures and Tables

**Table 1:** Characteristics of the population of Wales and HealthWise Wales participants.

Demographic Variable	Population of Wales	HealthWise Wales Participants ^1^
*Gender* ^2^	Female = 51%	Female = 75%
	Male = 49%	Male = 25%
*Age* (*in years*) ^3^		
16–24	11%	12%
25–44	24%	30%
45–64	26%	41%
>65	21%	16%
*Occupational Class* ^4^		
Professional	27%	50%
Intermediate	21%	18%
Routine and Manual	37%	11%
Other	15%	21%

^1^ Information taken from: HealthWise Wales: Resource Access Guidance for Researchers [21]. The characteristics of HealthWise Wales participants presented in Table 1 are those of the first 10,000 participants recruited to the platform. ^2^ Gender categories for other/prefer not to say were also available, but responses for these were too small to report. The gender breakdown presented is for persons aged 16 years and older to aid comparison with HealthWise Wales participants [22]. ^3^ Mid-year estimates for 2019 used for breakdown of population by age for Wales [22]. ^4^ Occupational Class breakdowns for Wales and HealthWise Wales participants taken from HealthWise Wales: Resource Access Guidance for Researchers [21]. Occupational classes come from the National Statistics Socio-economic Classification (NS-SEC) [23]. Professional occupations include higher managerial, higher administrative and traditional professional occupations. Intermediate occupations include secretary, personal assistant, clerical worker, office clerk. Routine and manual occupations include HGV driver, van driver, cleaner, porter, packer, sewing machinist, messenger, labourer and waiter/waitress.

**Table 2:** Participant demographic characteristics (*n* = 5584 unless otherwise stated ^1^).

Demographic Variable	*n* (%)
*Gender*	Female = 3877 (69.5)
	Male = 1703 (30.5)
	Other/Prefer not to say = 4 (<0.001)
*Age* (*in years*)	
16–24	288 (5.2)
25–44	1411 (25.3)
45–64	2273 (40.7)
>65	1610 (28.8)
*Ethnicity*	*n* = 5107; *missing data* = 477
Welsh	2921 (57.2)
Other British	1979 (38.8)
Irish	43 (0.8)
Other White background	89 (1.7)
Mixed/Multiple ethnic background	39 (0.7)
Asian background	<27 (0.7)
Black/African/Caribbean ethnic background	<15 (0.1)
Arab and other ethnic group	<16 (0.2)
*University Health Board* (*UHB*)	*n* = 5458; *missing data* = 126
Cardiff and Vale	1053 (18.9)
Aneurin Bevan	816 (14.6)
Abertawe Bro Morgannwg ^2^	780 (14.0)
Cwm Taf ^2^	750 (13.4)
Betsi Cadwaladr	738 (13.2)
Powys ^3^	705 (12.6)
Hywel Dda	616 (11.0)
*Level of Employment*	*n* = 5180; *missing data* = 404
Higher occupations	2659 (51.2)
Intermediate occupations	974 (18.8)
Lower occupations	489 (9.4)
Students or long-term unemployment	1058 (20.6)
*Level of deprivation*	*n* = 5458; *missing data* = 126
1—Most deprived	617 (11.3)
2	883 (16.2)
3	1107 (20.3)
4	1423 (26.1)
5—Least deprived	1428 (26.2)
*Urban and rural classification*	*n* = 5458; *missing data* = 126
Urban > 10 k	3290 (60.3)
Town and fringe	1018 (18.7)
Village, hamlet and isolated dwellings	1150 (21.1)
*Currently prescribed one or more medicines regularly* (*repeat prescription*) *by your doctor* (*Question 3*)	*n* = 5555; *missing data* = 29
	3733 (67.2)

^1^ Missing data are quantified in individual sections as the HealthWise Wales platform permits participants to submit incomplete questionnaires, both in core modules and researcher-led modules (such as this). ^2^ These two UHBs have been restructured since this study was undertaken. ^3^ Powys is a Teaching Health Board, not a University Health Board.

**Table 3:** Concerns about medicinal waste (*n* = 5573).

	Strongly Agree *n* (%)	Agree *n* (%)	Neither Agree nor Disagree *n* (%)	Disagree *n* (%)	Strongly Disagree *n* (%)	Don’t Know *n* (%)
Q4. I am concerned by the amount of prescription medicines which are wasted in the NHS	3143 (56.3)	1826 (32.8)	428 (7.7)	67 (1.2)	18 (0.3)	91 (1.6)

**Table 4:** Storage, disposal and return of medicines (*n* = 5584).

Question	Response Option	*n* = (%)
Q5. In your home, where do you store medicines that have been prescribed for you? (*Can select more than one option*)	*Kitchen*	2775 (49.7)
	*Bedroom*	1601 (28.7)
	*Bathroom*	1094 (19.6)
	*Living room*	244 (4.4)
	*Entrance hall*	25 (0.4)
	***Other***	
	*specific cupboard/cabinet/box or drawer*	39 (0.7)
	*utility/laundry room*	24 (0.4)
	*handbag, gym or work bag*	12 (0.2)
	*upstairs landing*	10 (0.2)
	*under the stairs*	8 (0.1)
	*dining room*	7 (0.1)
	*fridge*	7 (0.1)
	*hallway*	6 (0.1)
	*study or home office*	6 (0.1)
	*store room*	5 (0.1)
	*larder/pantry*	5 (0.1)
Q6. What do you do with prescription medicines that you no longer need? (*Can select more than one option*)	*Return to a pharmacy*	3032 (54.3)
	*Keep just in case I need in future*	1492 (26.7)
	*Throw out with household waste*	759 (13.6)
	*Return to GP*	134 (2.4)
	*Other*	365 (6.5)
Q7. What do you think currently happens to prescription medicines that are returned unused to the community pharmacy?	*Destroyed*	4330 (77.7)
	*Not sure*	979 (17.6)
	*Re-dispensed to other people*	129 (2.3)
	*Sent to developing countries or third world*	125 (2.2)
	*Other*	7 (0.1)

**Table 5:** Formulation of prescription medicine preparation accepted for re-dispensing.

Which of the Following Types of Prescription Medicines Would You Accept If They Were Re-Dispensed? (Question 8)	No *n* = (%)	Yes *n* = (%)	Unsure *n* = (%)
Tablet	647 (11.7)	4371 (78.7) *	533 (9.6)
Capsule	792 (14.3)	4147 (75.1) *	583 (10.6)
Skin patch	1852 (33.9)	2710 (49.6)	904 (16.5)
Liquid	2731 (50.0) *	1568 (28.7)	1164 (21.3)
Cream/ointment	2387 (43.6)	2113 (38.6)	971 (17.7)
Ear drop	2469 (45.2)	2033 (37.2)	956 (17.5)
Injection	2558 (46.8)	1848 (33.8)	1065 (19.5)
Eye drop	2723 (49.7)	1801 (32.8)	960 (17.5)
Nasal spray	2704 (49.4)	1763 (32.2)	1002 (18.3)
Suppository **	2743 (50.1) *	1754 (32.1)	974 (17.8)
Pessary **	2776 (51.8) *	1517 (28.3)	1069 (19.9)
Inhaler	2791 (51.3) *	1533 (28.2)	1121 (20.6)

* >50% acceptance or otherwise in bold. ** These questions contained an explanation of these forms of medicine.

**Table 6:** Factors affecting acceptance for reuse.

If You Were Given a Prescription Medicine Which Had Been Returned to the Pharmacy by Someone Else (Question 9)	Essential	Desirable	Unsure	Not Needed
The medicine has been checked by a pharmacist (*n* = 5522)	5103 (92.4) *	281 (5.1)	89 (1.6)	49 (0.9)
The medicine is still ‘in date’ (*n* = 5534)	4914 (88.8) *	470 (8.5)	99 (1.8)	49 (0.9)
The medicine has been returned unopened (*n* = 5536)	4750 (85.8) *	610 (11)	104 (1.9)	72 (1.3)
The medicine has been returned with an intact tamper proof seal (*n* = *5514*)	3923 (71.1) *	1179 (21.4)	220 (4.0)	192 (3.5)
I am informed that I am receiving a re-dispensed medicine (*n* = 5522)	3393 (61.4)	1154 (20.9)	333 (6.0)	642 (11.6)
I have the opportunity to give my consent to receive a re-dispensed medicine (*n* = 5517)	3343 (60.6)	1249 (22.6)	315 (5.7)	610 (11.1)
None of the tablets or capsules in the blisters have been used (*n* = 5521)	3028 (54.9)	1527 (27.7)	374 (6.8)	584 (10.6)
The medicine has been returned in packaging that has not been damaged (*n* = 5532)	2861 (51.8)	1999 (36.2)	225 (4.1)	436 (7.9)
The packaging of the medicine has been cleaned (*n* = 5490)	2842 (51.8)	1709 (31.1)	545 (9.9)	394 (7.2)

* >70% essential or otherwise in bold.

**Table 7:** Intentions to change medicines disposal practices if prescription medicines start to be reused and current disposal practices.

	More Likely to Return to a Pharmacy	Less Likely to Return to a Pharmacy	Would Not Change How I Get Rid of Medicines
Q11a. If prescription medicines did start to be re-dispensed, would you be more or less likely to return your unused prescription medicines to a pharmacy?	3143 (56.3)	1826 (32.8)	428 (7.7)

**Table 8:** Concerns about the safety of re-dispensed medicines.

Statement	Agree or Strongly Agree *n* = (%)	Neither Agree or Disagree *n* = (%)	Disagree or Strongly Disagree *n* = (%)	Don’t Know *n* = (%)
Q10c—Returned medicines could have been tampered with (*n* = 5514)	3817 (69.2)	1110 (20.1)	402 (7.3)	246 (4.5)
Q10b—It is safe for other people to use medicines that I have returned (*n* = 5522) ^1^	3814 (69.1)	957 (17.3)	457 (8.3)	294 (5.3)
Q10f—Returned medicines may have not been stored hygienically (*n* = 5513)	3604 (65.4)	1212 (22.0)	424 (7.7)	273 (5.0)
Q10a—Medicine packs that have been returned partly used should be destroyed (*n* = 5513)	2633 (47.9)	1118 (20.2)	1545 (28.0)	217 (3.9)
Q10h—Pharmacists may use re-dispensed medicines as an opportunity to commit fraud by charging the NHS for ‘new’ medicines when a re-dispensed medicine has been used (*n* = 5525)	1434 (26.0)	1700 (30.8)	1671 (30.2)	720 (13.0)
Q10i—Re-dispensing medicines could spread disease (*n* = 5499)	1119 (20.4)	1558 (28.3)	2203 (41.1)	619 (11.3)
Q10e—Returned medicines may be ineffective (*n* = 5507)	909 (16.5)	1207 (21.9)	3033 (55.1)	358 (6.5)
Q10g—It is not safe for medicines that have been returned by other people to be re-dispensed (*n* = 5503)	827 (16.0)	1359 (24.7)	2916 (52.9)	401 (7.3)
Q10d—Returned medicines are not safe to be re-dispensed (*n* = 5513)	744 (13.3)	1430 (25.9)	2999 (55.7)	340 (6.2)

^1^ Item reverse scored for scaling.

## Data Availability

Restrictions apply to the availability of these data. Application for access to the data can be made to HealthWise Wales.

## Supplementary Materials

The following are available online at https://www.mdpi.com/article/10.3390/pharmacy9020077/s1, Supplementary Materials Data S1: Participant Medicines Reuse Questionnaire. Supplementary Materials Data S2: Factor analysis of 9 items measuring views about the safety of medicines reuse.
