# Supplementary material for: Public Attitudes towards Medicinal Waste and Medicines Reuse in a ‘Free Prescription’ Healthcare System
Source: Pharmacy (Basel). 2021 Apr 8;9(2):77. doi: 10.3390/pharmacy9020077 (PMC8167727; doi:10.3390/pharmacy9020077)
Supplement: Supplementary file 1 [file pharmacy-09-00077-s001.zip › pharmacy-1047597-supplementary materials/pharmacy-1047597-Supplementary Materials Data S1 .docx]

**Unused medicines: should they be re-dispensed for other people?**

Prescription medicines are the most frequently used treatment in healthcare today. Around £800 million is spent by the National Health Service (NHS) on prescription medicines each year in Wales.

Some of these medicines are collected from pharmacies and taken home but are not used. This can happen for a number of reasons. We would be grateful if you could complete the following survey to help us understand how you store and dispose of medicines in your home, what you think currently happens to unused medicines and what you think about the idea of unused medicines being dispensed to other people.

**About you**

1. Do you consider yourself to be working in a health care role?

- Yes
- No – Go to question 3

1. What is your role in health care?

- Nursing
- Medicine
- Pharmacy
- Midwifery
- Health visitors
- Directors and managers
- Public health specialists
- Allied health profession
- Academic teaching and research
- Environmental health professionals
- Engineering
- Human resources / personnel
- IT
- Finance
- Domestic service staff
- Building services
- Other – Please specify __________

**Use of medicines**

1. Are you currently prescribed medication regularly (on repeat prescription) by your doctor?

(This includes: tablets, capsules, creams, inhalers, drops, sprays, patches etc.)

- Yes
- No

**Beliefs about medicines waste**

1. Please indicate the extent to which you agree with the statement below:

|  | Strongly Agree | Agree | Neither agree or disagree | Disagree | Strongly  Disagree | Don’t know |
| --- | --- | --- | --- | --- | --- | --- |
| I am concerned by the amount of prescription medicines which are wasted in the NHS |  |  |  |  |  |  |

**Storage and disposal**

1. In your home, where do you store medicines that have been prescribed for you? (Can select more than one option)

- Living room
- Kitchen
- Bathroom
- Bedroom
- Entrance hall
- Other – Please specify __________
- I don’t have medicines

1. What do you do with prescription medicines that you no longer need? (Can select more than one option)

- Throw out with household waste
- Keep just in case I need in future
- Return to a pharmacy
- Return to GP
- I don’t use medicines
- Other – Please specify __________

1. What do you think currently happens to prescription medicines that

are returned unused to community pharmacies?

[NOTE: ‘Community pharmacies’ are those pharmacies (sometimes called ‘chemists’) which can be found on the ‘high street’ or in some supermarkets. Pharmacies located in hospitals are not community pharmacies].

- - Re-dispensed to other people
  - Sent to developing countries (or ‘third world’)
  - Destroyed
  - Not sure
  - Other – Please specify __________

**Re-dispensing unused prescription medicines**

Currently, all medicines which are returned to community pharmacies are destroyed.

Some people think that prescription medicines which are returned unused to community pharmacies could be ‘reused’ by being dispensed (or re-dispensed) to other people who require the medication in the United Kingdom (UK). There are no plans for this to happen in the UK at present, but we would like to know your thoughts about this idea.

1. Which of the following types of prescription medicine would you accept if they were re-dispensed?

|  | Yes | No | Unsure |
| --- | --- | --- | --- |
| Liquid medicines |  |  |  |
| Inhalers |  |  |  |
| Tablets |  |  |  |
| Capsules |  |  |  |
| Creams or ointments |  |  |  |
| Suppositories  (medicines that are inserted into the rectum) |  |  |  |
| Pessaries  (medicines that are inserted into the vagina) |  |  |  |
| Injections |  |  |  |
| Skin patches |  |  |  |
| Nasal sprays or nose drops |  |  |  |
| Eye drops/eye ointments |  |  |  |
| Ear drops |  |  |  |

| **Please include any comments that you would like to make regarding this question below:** |
| --- |

1. If you were to be given a prescription medicine which had been returned to the pharmacy by someone else, what factors would be important to you in deciding whether or not you would be happy to accept it?

|  | Essential | Desirable | Unsure | Not needed |
| --- | --- | --- | --- | --- |
|  |  |  |  |  |
| The medicine has been returned unopened |  |  |  |  |
| The medicine is still ‘in date’ |  |  |  |  |
| The medicines has been returned in packaging that has not been damaged |  |  |  |  |
| None of the tablets or capsules in the blisters have been used |  |  |  |  |
| The medicine has been checked by a pharmacist |  |  |  |  |
| The packaging of the medicine has been cleaned |  |  |  |  |
| The medicine has been returned with an intact tamper proof seal |  |  |  |  |
| I am informed that I am receiving a re-dispensed medicine |  |  |  |  |
| I have the opportunity to give my consent to receive a re-dispensed medicine |  |  |  |  |

| **Please include any comments that you would like to make regarding this question below:** |
| --- |

1. When thinking about the idea of re-dispensing prescription medicines in general, to what extent do

you agree or disagree with the following statements?:

|  | Strongly Agree | Agree | Neither agree or disagree | Disagree | Strongly  Disagree | Don’t know |
| --- | --- | --- | --- | --- | --- | --- |
| Medicine packs that have been returned partly used should be destroyed |  |  |  |  |  |  |
| It is safe for other people to use medicines that I have returned |  |  |  |  |  |  |
| Returned medicines could have been tampered with |  |  |  |  |  |  |
| Returned medicines are not safe to be re-dispensed |  |  |  |  |  |  |
| Returned medicines may be ineffective |  |  |  |  |  |  |
| Returned medicines may have not been stored hygienically |  |  |  |  |  |  |
| It is not safe for medicines that have been returned by other people to be re-dispensed |  |  |  |  |  |  |
| Pharmacists may use re-dispensed medicines as an opportunity to commit fraud by charging the NHS for ‘new’ medicines when a re-dispensed medicine has been used |  |  |  |  |  |  |
| Re-dispensing medicines could spread disease |  |  |  |  |  |  |

| **Please include any comments that you would like to make regarding this question below:** |
| --- |

1. If prescriptions medicines did start to be re-dispensed. Would you be more or less likely to return your

unused prescription medicines to a pharmacy?

1. ● More likely to return to a pharmacy

● Less likely to return to a pharmacy

● Would not change how I get rid of medicines

1. How often do you return your unused medicines back to the pharmacy?

- Always
- Often
- Sometimes
- Rarely
- Never

Prescription medicines can cost the NHS anywhere between a few pounds to several thousand pounds.

1. If prescription medicines were to be re-dispensed, do you think that all medicines should be

considered for re-dispensing or only those which were expensive?

Please select one:

- - Only expensive medicines (perhaps costing the NHS greater than £20) should be considered for re-dispensing
  - All medicines should be considered for re-dispensing
  - Not sure

Please include any additional comments that you would like to make on the potential for unused prescription medicines to be re-dispensed to other people below:

|  |
| --- |

***PLEASE READ***

Please note that there are no plans for unused medicines to be re-dispensed to other people in the UK at this time. If you believe that this practice is happening at a pharmacy which you have used, please report your suspicion to the General Pharmaceutical Council.

Medicines which are no longer needed should be returned to your pharmacy for safe disposal.

If you have any questions about the medicines you are currently taking, please ask your pharmacist.

**Thank you for completing this questionnaire**
