# Supplementary material for: Public Attitudes towards Medicinal Waste and Medicines Reuse in a ‘Free Prescription’ Healthcare System
Source: Pharmacy (Basel). 2021 Apr 8;9(2):77. doi: 10.3390/pharmacy9020077 (PMC8167727; doi:10.3390/pharmacy9020077)
Supplement: Supplementary file 1 [file pharmacy-09-00077-s001.zip › pharmacy-1047597-supplementary materials/pharmacy-1047597-Supplementary Materials Data S2.docx]

**Supplementary Material 2: Factor analysis of 9 items measuring views about the safety of medicines reuse.**

**Table 1: Component Matrix - Principal Component Analysis for Questions 10a to 10i (n=5,584)**

|  | Component 1 | Component 2 |
| --- | --- | --- |
| Q10a  Q10b_reverse  Q10c  Q10d  Q10e  Q10f  Q10g  Q10h  Q10i | **0.572**  **0.524**  0.648  **0.844**  **0.769**  0.672  **0.854**  0.502  0.524 | -0.257  -0.557  **0.380**  -0.212  -0.116  **0.353**  -0.197  **0.506**  **0.179** |

2 components extracted. Total cumulative variance explained by the 2 factors = 59.779.

**Table 2: Rotated Component Matrix - Principal Component Analysis for Questions 10a to 10i (n=5,584)**

|  | Component 1 | Component 2 |
| --- | --- | --- |
| Q10a  Q10b_reverse  Q10c  Q10d  Q10e  Q10f  Q10g  Q10h  Q10i | **0.597**  **0.762**  0.226  **0.769**  **0.648**  0.262  **0.766**  0.033  0.451 | 0.193  -0.062  **0.717**  -0.408  -0.429  **0.713**  0.426  **0.712**  **0.648** |

Rotation methods – Varimax with Kaiser Normalisation

Items in bold are correlated and therefore form the two-factor structure.

**Table 3: Component Transformation Matrix -** **Principal Component Analysis for Questions 10a to 10i (n=5,584)**

| **Component** | **1** | **2** |
| --- | --- | --- |
| **1** | 0.742 | 0.670 |
| **2** | -0.670 | 0.742 |
